# Identification of selection signatures involved in performance traits in a paternal broiler line

**DOI:** 10.1186/s12864-019-5811-1

**Published:** 2019-06-03

**Authors:** Octávio Augusto Costa Almeida, Gabriel Costa Monteiro Moreira, Fernanda Marcondes Rezende, Clarissa Boschiero, Jane de Oliveira Peixoto, Adriana Mercia Guaratini Ibelli, Mônica Corrêa Ledur, Francisco José de Novais, Luiz Lehmann Coutinho

**Affiliations:** 1 University of São Paulo (USP) / Luiz de Queiroz College of Agriculture (ESALQ), Piracicaba, São Paulo Brazil; 2 Universidade Federal de Uberlândia (UFU), Uberlândia, Minas Gerais Brazil; 3 Noble Research Institute, Ardmore, OK USA; 4 Embrapa Suínos e Aves, Concórdia, Santa Catarina Brazil

**Keywords:** Runs of homozygosity, Fixation index, F_ST_, Artificial selection, *Gallus gallus*

## Abstract

**Background:**

Natural and artificial selection leads to changes in certain regions of the genome resulting in selection signatures that can reveal genes associated with the selected traits. Selection signatures may be identified using different methodologies, of which some are based on detecting contiguous sequences of homozygous identical-by-descent haplotypes, called runs of homozygosity (ROH), or estimating fixation index (F_ST_) of genomic windows that indicates genetic differentiation. This study aimed to identify selection signatures in a paternal broiler TT line at generations 7th and 16th of selection and to investigate the genes annotated in these regions as well as the biological pathways involved. For such purpose, ROH and F_ST_-based analysis were performed using whole genome sequence of twenty-eight chickens from two different generations.

**Results:**

ROH analysis identified homozygous regions of short and moderate size. Analysis of ROH patterns revealed regions commonly shared among animals and changes in ROH abundance and size between the two generations. Results also suggest that whole genome sequencing (WGS) outperforms SNPchip data avoiding overestimation of ROH size and underestimation of ROH number; however, sequencing costs can limited the number of animals analyzed. F_ST_-based analysis revealed genetic differentiation in several genomic windows. Annotation of the consensus regions of ROH and F_ST_ windows revealed new and previously identified genes associated with traits of economic interest, such as *APOB, IGF1, IGFBP2, POMC, PPARG,* and *ZNF423*. Over-representation analysis of the genes resulted in biological terms of skeletal muscle, matrilin proteins, adipose tissue, hyperglycemia, diabetes, *Salmonella* infections and tyrosine.

**Conclusions:**

Identification of ROH and F_ST_-based analyses revealed selection signatures in TT line and genes that have important role in traits of economic interest. Changes in the genome of the chickens were observed between the 7th and 16th generations showing that ancient and recent selection in TT line may have acted over genomic regions affecting diseases and performance traits.

**Electronic supplementary material:**

The online version of this article (10.1186/s12864-019-5811-1) contains supplementary material, which is available to authorized users.

## Background

Artificial selection of animals lead to changes on particular genomic regions that affect traits of economic interest, as well as traits involved in adaptation to climatic and stress conditions, immune response, and disease resistance [1]. Thus, selection signature regions are printed along the genome as a result of selection pressure. Detecting selection signatures is important for a better understanding of population history and genetic mechanisms affecting phenotypic differentiation in humans, livestock and wild animals [2]. Understanding how selection acts on livestock populations may also benefit breeding programs in order to improve traits of economic interest in these animals, such as chicken breeds which have been intensively selected for fast growth and muscle development [3]. Detection approaches rely on scanning the genome for regions of homozygosity, as well as on estimating allele or haplotype frequency differences between populations or generations within a population. There are several statistical methods for these analyses, such as extended haplotype homozygosity (EHH) [4], integrated haplotype score (iHS) [5], runs of homozygosity (ROH) [2], and F_ST_ statistics [6].

Runs of homozygosity are regions in the genome containing contiguous homozygous genotypes identical by descent (IBD), i.e. regions where the pairs of alleles are most likely inherited from a common ancestor [2]. Recent studies used this approach to better understand human diseases [7–9], human ancestry [10], and population structure and traits of interest in livestock species, such as cattle [11–14], swine [15], poultry [16, 17], and sheep [18]. The fixation index (F_ST_), first defined by Wright [19], is a measure that exploits differences in allele frequencies to infer the genetic differentiation between populations or generations [20]. A certain locus under selection pressure changes its frequency over the generations. Thus, high values of F_ST_ indicate candidate selection signatures due to differences in locus frequency among populations or across generations. Previous studies have reported important selection signatures in Virginia [21] and Brazilian broiler and layer chicken lines [22] using this method.

Embrapa Swine and Poultry, a Brazilian National Research Center, has been raising experimental chicken populations under selection since the 1970’s. One of these lines is the paternal broiler line called TT, which has been under multi-trait selection since 1992 [23]. Identification of selection signatures in chicken lines can help understand which regions underwent selection pressure over time and how their biological mechanisms act to express the traits of interest, such as muscle growth and fat deposition. In this sense, we aimed to investigate selection signatures in TT broiler line by detecting ROH in the 7th and 16th generations, raised in the years of 1998 and 2007, respectively, and estimating F_ST_ statistic between these two generations. The identification of those regions will provide better understanding of artificial selection effects on broiler lines, and may point out candidate genes and biological mechanisms underlying performance traits.

## Methods

### Ethics statement

This study followed experimental protocols pertinent to animal experimentation with the approval of the Embrapa Swine and Poultry Ethics Committee on Animal Utilization (CEUA) in Concordia, Santa Catarina State, Brazil, on resolution number 011/2010. It followed the rules of National Council of Animal Experimentation Control (CONCEA) in accordance with international guidelines to guarantee animal welfare.

### Chicken population

Chickens used in this study were from a broiler line developed by the Embrapa Swine and Poultry National Research Center. This line, called TT, was originated from Cornish and White Plymouth Rock breeds, that has been under a multi-trait selection process focused on body weight, feed conversion, cut yields, breast weight, abdominal fat, and other traits, since 1992 [17, 23, 24]. The chickens were raised in open sided poultry houses, receiving commercial broiler diet and water ad libitum [17, 24]. Chickens were euthanized by cervical dislocation at 42 days of age. Two groups of animals from this line were analyzed, 14 chickens (half male and half female) from the seventh-generation (7th) raised in the year of 1998 and 14 male chickens from the sixteenth-generation (16th) raised in 2007.

The performance of birds from the 7th generation, as hatched average live weight at 35 days of age was 2272 g; the breast area in the live bird was 96,1 cm^2^ and the individual feed conversion rate (FCR) for males, from 36 to 43 days of age was 2268 g. The performance of birds from 16th, as hatched average live weight at 42 days of age was 2457 g; the breast area was 112 cm^2^ and the individual FCR from 43 to 49 days of age was 2798 g [17]. Note that the age of selection for BW and BA has changed from 35 to 42 days of age in the described period, as well as the FCR evaluation period, which has changed from 36 to 43 days in 1998 to 43–49 days of age in 2007.

### Sequencing and quality control

Whole genome sequencing (WGS) data of 28 chickens were used in this study. Animals were individually sequenced to a minimum coverage of 11.4x using the HiSeq2500 (Illumina) platform, and the alignment of reads was done against the chicken genome assembly (Gallus_gallus-5.0, UCSC) chicken reference genome using Bowtie2 [25]. Detailed information about library preparation, sequencing, quality control of reads, alignment and SNP and INDEL identification are fully described in Boschiero et al. [22] and Moreira et al. [26]. Variants identified in sexual, mitochondrial, random or unplaced chromosomes were removed from our analysis.

### Principal component analysis

Genetic relationship between the 28 animals was assessed with a principal component analysis (PCA) using the SNP dataset (*n* = 9,914,904). The analysis was performed using the SNPRelate package of Bioconductor by means of an in-house script in R.

### Identification of runs of homozygosity

The identification of ROH was chosen to obtain information about selection signatures and how they are shared between animals in both generations. Analyses were performed using PLINK v1.9 software [27, 28], which uses a sliding window approach: a window, with a minimum size, slides across the genome, calling a segment if it is in accordance with the parameters established and the threshold of calculated proportion of homozygous windows overlapping each SNP in that segment. The parameters used in the analysis were set based on Ceballos et al. [29] and they are listed in Table 1.

**Table 1:** PLINK parameters for run of homozygosity (ROH) analysis

Parameter	Value	Definition
-homozyg-snp	50	Minimum number of SNP required to consider a ROH;
-homozyg-kb	300	Size (Kb) of the sliding window;
-homozyg-density	50	Minimum density required to consider a ROH;
-homozyg-gap	1000	Maximum size (Kb) between two SNP to be considered in the same ROH;
-homozyg-window-snp	50	Number of SNP present in the sliding window;
-homozyg-window-het	3	Number of heterozygous SNP allowed in a ROH;
-homozyg-window-missing	5	Number of missing calls allowed in a ROH;
-homozyg-window-threshold	0.05	Proportion of overlapping windows that must be called homozygous to define a given SNP as in a homozygous segment.

Dataset of the 28 animals comprised 9,914,904 SNP, and all INDEL were excluded. The parameter *-homozyg-group* was also used to obtain information of the overlapping ROH (pools), i.e., ROH that appeared in at least two animals in the same region of the genome. The output *plink.hom.overlap* shows each ROH of each animal overlapping and their respective union (uROH) and consensus sequences (cROH), besides their genome position, size and number of SNP. The consensus ROH (cROH, i.e. a consensus segment of ROH that appeared in a common region in at least two animals) of the pools were used for annotation and enrichment analysis, to avoid randomly assigned ROH and to represent what changed and what is conserved between the animals [30, 31]. In addition, we used an in-house script in R to check the overlap between the regions of all cROH and the ROH previously identified in the TT Reference population (originated from TT broiler line) by Marchesi et al. [17], given the positional coordinates (chromosome, start and end) of these regions and considering at least one overlapped base pair.

### Genomic inbreeding coefficients

Individual genomic inbreeding coefficients were calculated based on ROH data (F_ROH_), as defined by McQuillan et al. [32], to know if there was a difference of inbreeding between the 7th and 16th generations. F_ROH_ was calculated as:

$$ {F}_{ROH}=\frac{L_{ROH}}{L_{aut}}, $$

where L_ROH_ is the total size of ROH in the genome and L_aut_ is the total size of autosomal genome covered by SNP of an individual (933.071 Mb, Gallus_gallus-5.0 chicken reference genome - UCSC).

### F_ST_ analysis

This method was applied to compare the two generations, i.e. to identify selection signatures by estimating the differences in allele frequency between the 7th and 16th generations. The fixation index was calculated according to Weir and Cockerham’s pairwise estimator method [33] using VCFtools v.1.16 software [34], in which SNP and INDEL analyses were run separately, comprising datasets of 9,914,904 SNP and 793,603 INDEL. The same parameters used recently in chickens by Boschiero et al. [22] were applied: F_ST_ values were calculated using overlapping windows of 20 Kb size sliding by steps of 10 Kb size. Windows with less than 10 SNP or 5 INDEL were removed, and all negative values were set to zero. F_ST_ values of the remaining windows were ranked, and those equal or above 0.3 were considered as candidate selection signatures. The software BEDTools [35] was used to check if there were equivalent regions identified in both datasets.

### Functional analysis

Functional analysis was performed to identify genes annotated within the candidate selection signature regions identified and, consequently, the biological mechanisms that may be involved with traits of adaptation and performance. Such information was obtained assessing the position (start and end coordinates) of the candidate selection signatures (cROH and FST windows ≥0.3) in the chicken genome available at BioMart Ensembl genome browser platform (Ensembl Genes release 94, Gallus_gallus-5.0 assembly) [36].

We also assessed the genes annotated in the candidate selection signatures under different perspectives in order to understand the effects of selection on TT line in different periods. First, we investigated changes that occurred between the 7th and 16th generations: (i) cROH of regions shared exclusively among animals of the 7th; (ii) cROH of regions shared exclusively among animals of the 16th; (iii) cROH of regions that were shared among at least four animals of the 16th more than animals of 7th; (iv) FST SNP windows (≥ 0.3); and (v) FST INDEL windows (≥ 0.3). In addition, we looked for genes annotated in (vi) cROH of regions shared with 12 or more animals (among the 28), to identify regions probably related to chicken domestication or even, specialization into broilers.

Functional enrichment was performed using MeSH Enrichment and Semantic Analysis, Bioconductor’s package [37, 38], in R software [39] to investigate if there was overrepresentation of any biological processes and components. For such purpose, datasets of genes annotated in the specific candidate selection signatures previously mentioned (i-vi) were analyzed separately. The *p-value* was adjusted using the Benjamin-Hochberg false discovery rate (FDR) method [40].

### Overlapping selection signatures with QTL

In order to confirm the role of the selection signatures detected herein in the regulation of important phenotypes in chickens, we investigated the overlap with QTL associated with traits of economic interest. The analysis was performed using an in-house script in R to overlap the regions of all cROH, F_ST_ SNP and INDEL windows against the QTLs available at the Chicken QTL database [41], given the positions (chromosome, start and end) of these regions and considering at least one overlapped base pair. Particularly, we also analyzed if there was overlap of candidate selection signatures with QTL associated with fat deposition previously identified by Moreira et al. [42] in the TT Reference population, originated from an expansion of TT line in 2007 for genomic studies purpose [17].

## Results

### Principal component analysis

**Fig. 1:**
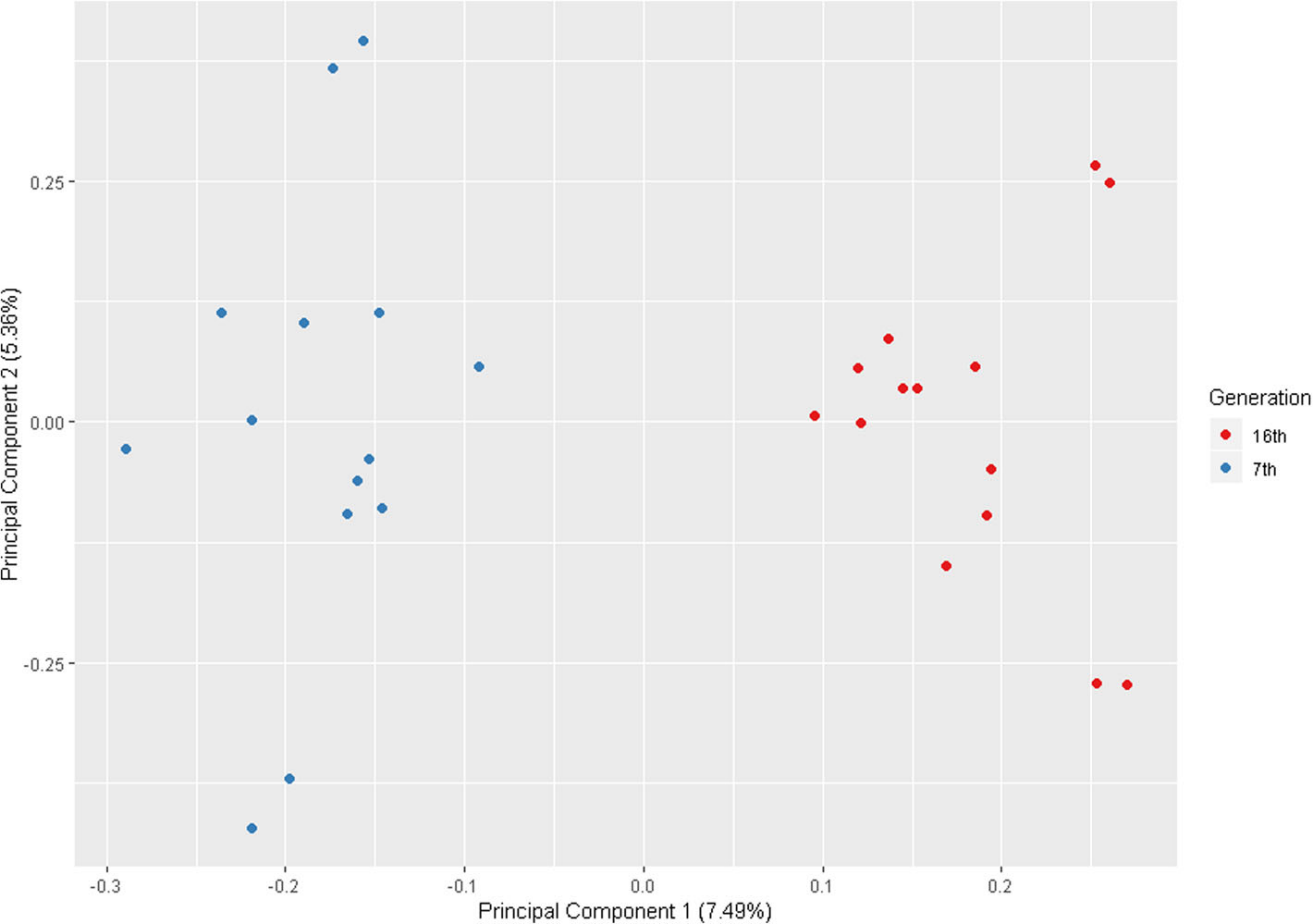
Principal component analysis using genomic data (SNP = 9,914,904) for 7th and 16th generations

Principal component analysis using genomic data revealed a cluster separation between animals of the 7th and the 16th generations (Fig. 1). The distinct clustering demonstrated that genome data successfully separate these animals accordingly to their generation.

### Runs of homozygosity

**Fig. 2:**
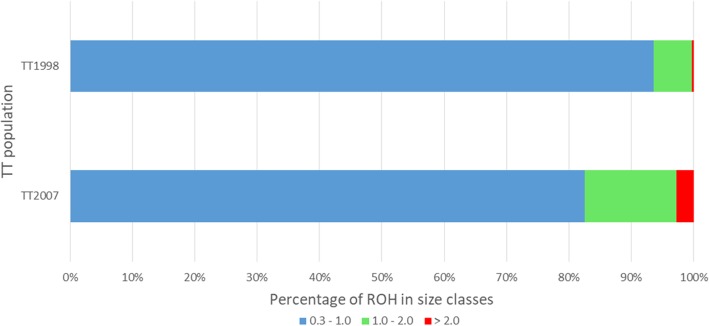
Percentage of ROH in 7th and 16th generations distributed in size classes. A decrease in the proportion of ROH smaller than 1.0 Mb in 16th is observed, meanwhile the proportions of ROH with sizes above 1.0 MB increased

Analysis of the whole genome sequence data of the 28 animals (14 of the 7th and 14 of the 16th generation) with PLINK’s sliding window approach identified 5721 ROH (1944 in the 7th and 3777 in the 16th generation animals) (Additional file 1). The average number of segments per animal was lower in the 7th (138.9 ROH/animal) than in the 16th generation (269.8 ROH/animal) (Table 2). The ROH presented small and moderate sizes, ranging between 300 Kb and 4.9 Mb, and most of them had sizes smaller than 1.0 Mb in both generations (1821 ROH in the 7th and 3120 in the 16th generation animals). A change in the distribution of ROH sizes was also observed between both generations (Fig. 2). The proportion of ROH smaller than 1.0 Mb decreased (93.7% in the 7th to 82.6% in 16th generation) while the proportion of ROH with sizes between 1.0 and 2.0 Mb increased (6.1% in the 7th to 14.7% in the 16^th^generation) as well as ROH bigger than 2.0 Mb (0.3% in the 7th to 2.7% in 16th generation). The average total size of ROH per animal was 73.2 Mb in the 7th and 188.6 Mb in the 16th generation (Table 2).

**Table 2:** ROH features for each animal of 7th and 16th generations

IID	Number of ROH^a^	Total size^b^ (Kb)	Mean size of segments (Kb)	F_ROH_
7th_01	130	60,492.6	465.328	0.0648
7^th^_02	212	125,062.0	589.913	0.1340
7^th^_03	131	63,732.1	486.504	0.0683
7^th^_04	61	25,416.8	416.668	0.0272
7^th^_05	65	30,014.6	461.763	0.0322
7^th^_06	85	38,303.9	450.634	0.0411
7^th^_07	183	103,302.0	564.491	0.1107
7^th^_08	148	71,381.7	482.309	0.0765
7^th^_09	209	138,608.0	663.195	0.1486
7^th^_10	219	128,120.0	585.024	0.1373
7^th^_11	94	42,055.7	447.401	0.0451
7^th^_12	175	82,420.5	470.974	0.0883
7^th^_13	188	95,302.7	506.929	0.1021
7^th^_14	44	20,107.3	456.985	0.0215
Means (CV) of the 7^th^ generation	139 (43.8%)	73,165.7 (54.8%)	503,437 (13.9%)	0,0784 (54.8%)
16th_01	245	164,355.0	670.839	0.1761
16^th^_02	241	183,336.0	760.729	0.1965
16^th^_03	254	174,906.0	688.608	0.1875
16^th^_04	280	200,919.0	717.567	0.2153
16^th^_05	268	188,023.0	701.579	0.2015
16^th^_06	318	200,111.0	629.281	0.2145
16^th^_07	256	181,422.0	708.681	0.1944
16^th^_08	290	196,574.0	677.843	0.2107
16^th^_09	289	206,456.0	714.381	0.2213
16^th^_10	283	215,635.0	761.963	0.2311
16^th^_11	275	187,931.0	683.386	0.2014
16^th^_12	254	181,634.0	715.095	0.1947
16^th^_13	242	167,875.0	693.697	0.1799
16^th^_14	282	191,333.0	678.484	0.2051
Means (CV) of the 16^th^ generation	270 (8.3%)	188,607.9 (7.7%)	700.152 (4.9%)	0.2021 (7.7%)

*IID* individual identification, *CV* coefficient of variation %

^a^Total number of ROH identified in each animal

^b^Total size of autosomal genome covered by ROH

**Fig. 3:**
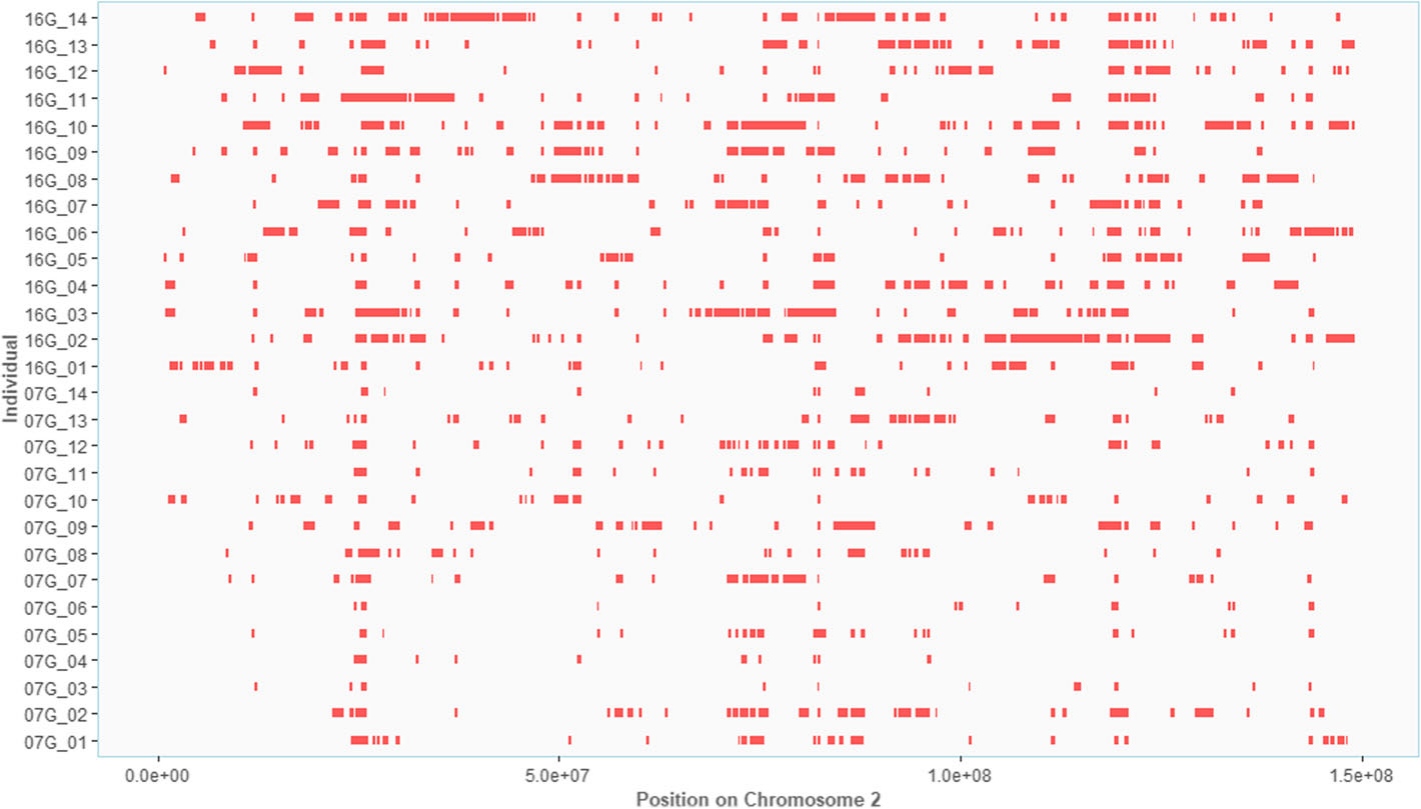
Genome wide distribution of runs of homozygosity (ROH) in TT population. Size and location of ROH in chromosome 2 for each animal are represented in parallel. Patterns of shared ROH can be observed in some regions of the chromosome. In addition, a higher frequency of ROH in animals of 16G was observed

ROH were identified in all chromosomes, except on GGA16 and GGA30–32 (Table 3). Figure 3 represents all ROH, with their proportional sizes, distributed across GGA2 for the 28 animals. Regions where ROH is presented in more than one animal formed a sharing pattern. Figures of other chromosomes are provided as Additional file 2. The four longest ROH (> 4 Mb) were located on different macro chromosomes (GGA2: 116,060,874 – 120,088,450; GGA5: 39,097,092 – 43,183,508; GGA3: 25,504,098 – 29,639,462; GGA4: 69,071,022 – 73,960,022). Overlaps of ROH from at least two animals established 1941 pools (Additional file 3). There was one pool of ROH shared among the 28 animals, and it was located in the GGA2 with a consensus sequence of 300.2 Kb (82,146,603 – 82,446,837). Furthermore, most of the pools consisted in regions shared among two to seven animals (74.4%). There were 87 regions with ROH commonly shared with at least 12 animals, and most of them identified on GGA1.

**Table 3:** Summary of runs of homozygosity (ROH), pools of ROH, F_ST_ SNP windows, and F_ST_ INDEL windows by chromosome in TT population in all animals from 7th and 16th generations

GGA	Size (Mb)	Number of ROH	Number of pools	Number of FST SNP windows (≥ 0.3)	Number of FST INDEL windows (≥ 0.3)
1	196.20	1230	415	53	65
2	149.56	1087	338	28	28
3	111.30	609	228	28	23
4	91.28	582	212	5	4
5	59.83	392	137	6	9
6	35.47	211	77	4	5
7	36.95	267	85	17	21
8	29.96	213	66	1	2
9	24.09	106	39	6	10
10	20.44	106	39	–	2
11	20.22	114	36	1	1
12	19.95	90	41	–	–
13	18.41	107	35	1	2
14	15.60	101	32	–	1
15	12.76	81	32	–	–
17	10.96	72	22	–	–
18	11.05	87	28	1	–
19	9.98	61	16	–	2
20	14.11	54	19	–	–
21	6.86	26	7	–	–
22	4.73	13	4	2	3
23	5.79	21	7	1	–
24	6.28	30	12	–	–
25	2.91	7	1	–	–
26	5.31	9	1	–	–
27	5.66	16	5	–	–
28	4.97	25	6	–	–
33	1.65	4	1	–	–

**Fig. 4:**
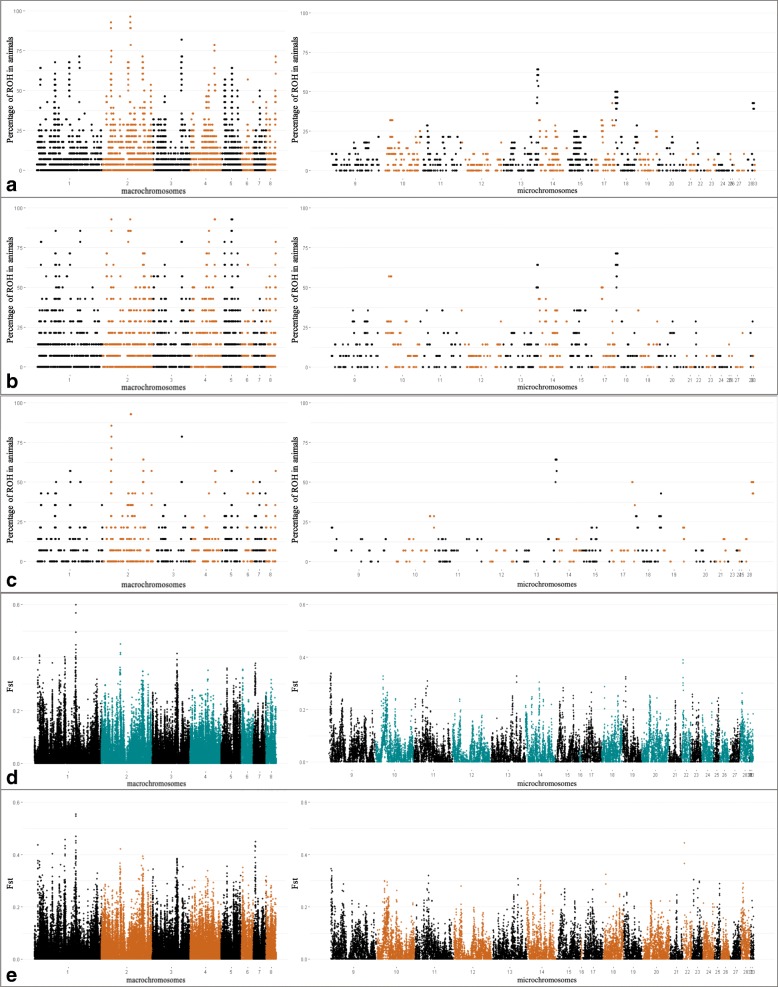
Manhattan plot of genomic regions and percentage of animals that shares the candidate selection signatures as well, SNPs and INDELs Fst windows. **a**: ROH pools detected in all the 28 animals; **b**: ROH pools detected in the 14 birds from 16th generation; **c**: ROH pools detected in the 14 birds from 7th generation; **d**: SNPs Fst windows; **e**: INDELS Fst windows. For **a**, **b** and **c**, the X-axis represents the chromosomes, and Y-axis shows the proportion of animals that shares the ROH pools. For **d** and **e**, the X-axis represents the chromosomes, and Y-axis shows the Fst values

Regions commonly shared among the animals become more frequent in the 16th generation. We observed a greater number of ROH pools shared among animals from 16th generation than animals from 7th generation (Fig. 4-a, b and c).

### Genomic inbreeding coefficients

Individual genomic inbreeding coefficients based on ROH (F_ROH_) were calculated for both generations (Table 2). Mean, maximum and minimum individual F_ROH_ for animals of the 7th generation were 0.0784, 0.1340, and 0.0215, respectively, with a coefficient of variation (CV) of 52.8%. For animals of the 16th, the mean, maximum and minimum individual F_ROH_ were 0.2021, 0.2213, and 0.1761 (CV = 7.4%).

### F_ST_ windows

F_ST_ analysis identified 91,638 and 86,404 windows for SNP and INDEL datasets, respectively, after removing windows with less than 10 SNP and five INDEL. The number of markers per window ranged from 10 to 1562 SNP (average of 216.3 SNP/window) and from five to 72 INDEL (average of 18.2 INDEL/window). Mean F_ST_ values for SNP and INDEL datasets were 0.040 and 0.038, respectively, while the highest F_ST_ values were 0.598 and 0.555.

**Fig. 5:**
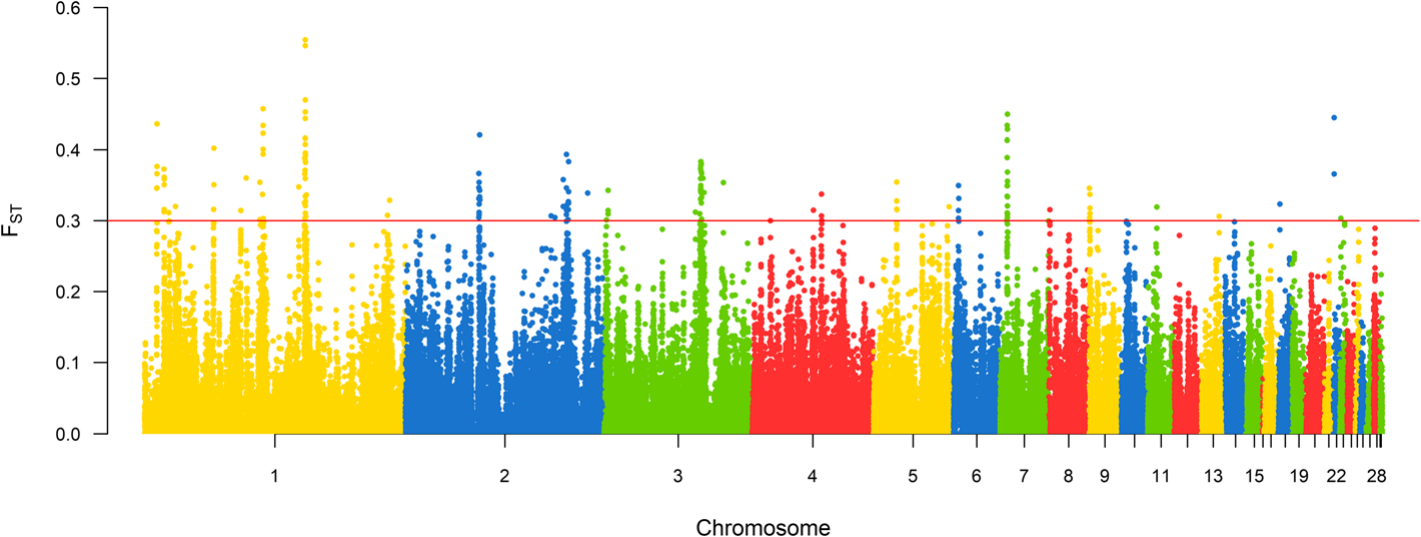
Manhattan plot of genome wide distribution of F_ST_ windows for SNP dataset. Red line represents threshold of 0.3, windows above this value were considered candidate selection signature

**Fig. 6:**
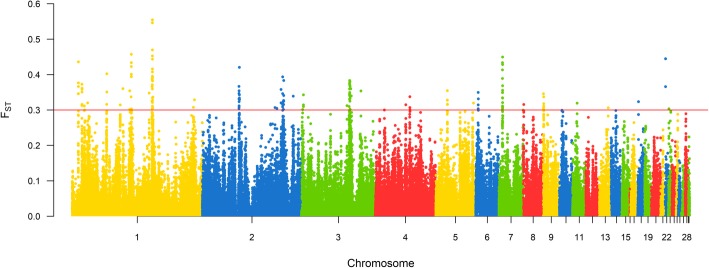
Manhattan plot of genome wide distribution of F_ST_ windows for INDEL dataset. Red line represents threshold of 0.3, windows above this value were considered candidate selection signatures

Windows with FST values equal or higher than 0.3 were considered candidate selection signatures. There were 178 windows using SNP dataset (Fig. 5) and 154 windows using INDEL dataset (Fig. 6) above this threshold value (FST ≥ 0.3). More information about these windows are available in the Additional files 5 and 6. Most of these windows were in the macrochromosomes (Table 3) and approximately 87% of the INDEL windows overlapped with SNP windows (Fig. 4 – c and d).

### Genes in consensus selection signature regions

Annotation analysis using the Ensembl genome browser revealed 5681 genes annotated in the 1941 cROH pools shared among at least two of the 28 animals (Additional file 7). Annotation of F_ST_ windows (Fst ≥0.3) identified 56 and 60 genes for SNP and INDEL datasets, respectively (Additional files 8 and 9). Since a great part of SNP windows overlapped with INDEL windows, 37 of these genes were common for both datasets (Table 4). In addition, about 46.1% of SNP F_ST_ windows and 37.7% of INDEL F_ST_ windows overlapped with cROH. Thus, we found 34 genes annotated in cROH in common with genes annotated in FST (SNP and/or INDEL) windows (Table 4).

**Table 4:** Genes annotated commonly between selection signatures of two or more datasets

Gene ID	Gene name	Description
*F*_*ST*_ *SNP and INDEL windows (≥0.3) datasets*		
ENSGALG00000000242	*EBF2*	early B cell factor 2
ENSGALG00000002370	*SH2D4B*	SH2 domain containing 4B
ENSGALG00000002414	*TSPAN14*	tetraspanin 14
ENSGALG00000004045	*AGAP1*	ArfGAP with GTPase domain, ankyrin repeat and PH domain 1
ENSGALG00000004116	*TRPM8*	transient receptor potential cation channel subfamily M member 8
ENSGALG00000004129	*SPP2*	secreted phosphoprotein 2
ENSGALG00000007555	*CCND1*	cyclin D1
ENSGALG00000007556	*LTO1*	LTO1, ABCE1 maturation factor
ENSGALG00000012542	*RASD2*	RASD family member 2
ENSGALG00000015402	*C3orf38*	chromosome 3 open reading frame 38
ENSGALG00000015403	*EPHA3*	EPH receptor A3
ENSGALG00000015570	*GPR63*	G protein-coupled receptor 63
ENSGALG00000015573	*FHL5*	four and a half LIM domains 5
ENSGALG00000016518	*PHKA2*	phosphorylase kinase regulatory subunit alpha 2
ENSGALG00000016522	*PPEF1*	protein phosphatase with EF-hand domain 1
ENSGALG00000016529	*CDKL5*	cyclin dependent kinase like 5
ENSGALG00000016541	novel gene	BEN domain containing 2
ENSGALG00000016543	*NHS*	NHS actin remodeling regulator
ENSGALG00000022866	*ZNF654*	zinc finger protein 654
ENSGALG00000026372	novel gene	--
ENSGALG00000028376	*FGF19*	fibroblast growth factor 19
ENSGALG00000032974	*ADAMTS2*	ADAM metallopeptidase with thrombospondin type 1 motif 2
ENSGALG00000033076	novel gene	--
ENSGALG00000035116	*STAG1*	stromal antigen 1
ENSGALG00000035393	*LRRC14B*	leucine rich repeat containing 14B
ENSGALG00000035906	*YTHDC1*	YTH domain containing 1
ENSGALG00000036204	novel gene	--
ENSGALG00000036327	*NGEF*	neuronal guanine nucleotide exchange
ENSGALG00000036730	*MRPS35*	mitochondrial ribosomal protein S35
ENSGALG00000036938	*RALYL*	RALY RNA binding protein like
ENSGALG00000038154	*YAP1*	Yes associated protein 1
ENSGALG00000038730	*GIGYF2*	GRB10 interacting GYF protein 2
ENSGALG00000039139	*TNS3*	tensin 3
ENSGALG00000039690	*STMN2*	stathmin 2
ENSGALG00000039738	*SLC9A3*	solute carrier family 9 member A3
ENSGALG00000040264	*C9H2ORF82*	chromosome 9 open reading frame, human C2orf82
ENSGALG00000042764	*COG5*	component of oligomeric golgi complex 5
*cROH and F*_*ST*_ *SNP windows (≥0.3)*		
ENSGALG00000000242	*EBF2*	early B cell factor 2
ENSGALG00000001153	*AUTS2*	AUTS2, activator of transcription and developmental regulator
ENSGALG00000003705	*VPS13C*	vacuolar protein sorting 13 homolog C
ENSGALG00000004045	*AGAP1*	ArfGAP with GTPase domain, ankyrin repeat and PH domain 1
ENSGALG00000004116	*TRPM8*	transient receptor potential cation channel subfamily M member 8
ENSGALG00000006237	*PKN2*	protein kinase N2
ENSGALG00000007555	*CCND1*	cyclin D1
ENSGALG00000007556	*LTO1*	LTO1, ABCE1 maturation factor
ENSGALG00000015402	*C3orf38*	chromosome 3 open reading frame 38
ENSGALG00000015403	*EPHA3*	EPH receptor A3
ENSGALG00000016518	*PHKA2*	phosphorylase kinase regulatory subunit alpha
ENSGALG00000016522	*PPEF1*	protein phosphatase with EF-hand domain 1
ENSGALG00000016529	*CDKL5*	cyclin dependent kinase like 5
ENSGALG00000022866	*ZNF654*	zinc finger protein 654
ENSGALG00000025253	*gga-mir-1694*	gga-mir-1694
ENSGALG00000028376	*FGF19*	fibroblast growth factor 19
ENSGALG00000030580	*RPS6KA5*	ribosomal protein S6 kinase A5
ENSGALG00000032958	*AMPH*	amphiphysin
ENSGALG00000034119	novel gene	collagen type XV alpha 1 chain
ENSGALG00000035116	*STAG1*	stromal antigen 1
ENSGALG00000035906	*YTHDC1*	YTH domain containing 1
ENSGALG00000036938	*RALYL*	RALY RNA binding protein like
ENSGALG00000038730	*GIGYF2*	GRB10 interacting GYF protein 2
ENSGALG00000040167	*TPD52*	tumor protein D52
*cROH and F*_*ST*_ *INDEL windows (≥0.3) datasets*		
ENSGALG00000000242	*EBF2*	early B cell factor 2
ENSGALG00000000667	*EDN2*	endothelin 2
ENSGALG00000004045	*AGAP1*	ArfGAP with GTPase domain, ankyrin repeat and PH domain 1
ENSGALG00000004116	*TRPM8*	transient receptor potential cation channel subfamily M member 8
ENSGALG00000007555	*CCND1*	cyclin D1
ENSGALG00000007556	*LTO1*	LTO1, ABCE1 maturation factor
ENSGALG00000008163	*PSME4*	proteasome activator subunit 4
ENSGALG00000015402	*C3orf38*	chromosome 3 open reading frame 38
ENSGALG00000015403	*EPHA3*	EPH receptor A3
ENSGALG00000016518	*PHKA2*	phosphorylase kinase regulatory subunit alpha
ENSGALG00000016522	*PPEF1*	protein phosphatase with EF-hand domain 1
ENSGALG00000016529	*CDKL5*	cyclin dependent kinase like 5
ENSGALG00000022866	*ZNF654*	zinc finger protein 654
ENSGALG00000025789	*gga-mir-6614*	gga-mir-6614
ENSGALG00000027632	*ACYP2*	acylphosphatase 2
ENSGALG00000027960	*GRPR*	gastrin releasing peptide receptor
ENSGALG00000028376	*FGF19*	fibroblast growth factor 19
ENSGALG00000034516	*SHISA6*	shisa family member 6
ENSGALG00000035116	*STAG1*	stromal antigen 1
ENSGALG00000035906	*YTHDC1*	YTH domain containing 1
ENSGALG00000036810	novel gene	--
ENSGALG00000036938	*RALYL*	RALY RNA binding protein like
ENSGALG00000038730	*GIGYF2*	GRB10 interacting GYF protein 2
ENSGALG00000039102	*TOX*	thymocyte selection associated high mobility
ENSGALG00000040322	novel gene	--
*cROH and F*_*ST*_ *SNP and INDEL windows (≥0.3) datasets*		
ENSGALG00000000242	*EBF2*	early B cell factor 2
ENSGALG00000004045	*AGAP1*	ArfGAP with GTPase domain, ankyrin repeat and PH domain 1
ENSGALG00000004116	*TRPM8*	transient receptor potential cation channel subfamily M member 8
ENSGALG00000007555	*CCND1*	cyclin D1
ENSGALG00000007556	*LTO1*	LTO1, ABCE1 maturation factor
ENSGALG00000015402	*C3orf38*	chromosome 3 open reading frame 38
ENSGALG00000015403	*EPHA3*	EPH receptor A3
ENSGALG00000016518	*PHKA2*	phosphorylase kinase regulatory subunit alpha
ENSGALG00000016522	*PPEF1*	protein phosphatase with EF-hand domain 1
ENSGALG00000016529	*CDKL5*	cyclin dependent kinase like 5
ENSGALG00000022866	*ZNF654*	zinc finger protein 654
ENSGALG00000028376	*FGF19*	fibroblast growth factor 19
ENSGALG00000035116	*STAG1*	stromal antigen 1
ENSGALG00000035906	*YTHDC1*	YTH domain containing 1
ENSGALG00000036938	*RALYL*	RALY RNA binding protein like
ENSGALG00000038730	*GIGYF2*	GRB10 interacting GYF protein 2

Based on Biomart Ensembl database, some of the genes commonly annotated in Fst and cROH regions, plays a role in biological processes involved in traits of economic interest in chicken or in other model animals; all the biological processes related to the genes mentioned in Table 4, are available at Additional file 10. There were genes involved in lipid metabolic processes, glucose metabolism and homeostasis and adipose tissue development. Other genes were described to be involved in muscle cell differentiation, muscle tissue development, and constituents of skeletal muscle. Moreover, there were a group of genes related to different types of behavior, such as grooming, locomotion, fear response, feeding behavior, aggressiveness, and social, exploration and maternal behaviors. Genes involved in the immune humoral system, differentiation, proliferation, homeostasis and chemotaxis of B cells, and regulation of cytokines production were also annotated in regions of the candidate selection signatures.

In order to complement the Fst analysis we identified genes in cROH regions that were either exclusive between the 7th and 16th generations. There were 71 genes annotated in the regions that were in homozygosity only in the 7th generation and 1881 genes annotated in regions of cROH shared only among animals of the 16th generation. We also identified genes in cROH regions that had changed between the 7th and 16th generations. For that, we considered regions that either increased or decreased by at least four animals in cROH regions between generations. There were 1318 genes annotated on these regions. For example, the gene *IGF-I* (GGA1 55,335,204 – 55,383,631) was annotated in a cROH region (GGA1 55,149,208 – 55,359,089) shared between seven animals of the 7th and 13 animals of the 16th. Additional file 11 presents genes annotated in these regions and that were previously associated with traits of economic interest in chickens..

These different gene lists were used to perform MeSH overrepresentation analysis, with the purpose of having an integrated knowledge of biological processes may be involved in the selection of TT line. MeSH analysis indicates if there is an overrepresentation of a particular group of genes in a biological category, such as anatomy, diseases or phenomena and processes. In this sense, overrepresentation analysis resulted in eight different biological terms: ‘matrilin proteins’, ‘skeletal muscle’, ‘*Salmonella* infections in animals’, ‘adipose tissue’, ‘cystatins’, ‘tyrosine’, ‘pregnancy in diabetics’, and ‘hyperglycemia’. Table 5 presents each one of these terms and their respective gene counts and *p*-values.

**Table 5:** MeSH enrichment analysis of genes annotated in candidate selection signatures

MeSH term (MeSH ID)	Gene count	*P-value*	BH^a^	Dataset^b^
Matrilin Proteins (D064235)	3	0.00008523	0.01605961	a
Muscle, Skeletal (D018482)	9	0.00037904	0.04927546	a
Salmonella Infections, Animal (D012481)	2	0.02170325	0.02893767	b
Adipose Tissue (D000273)	9	0.00246916	0.0157409	c
Cystatins (D015891)	2	0.00018075	0.01590614	d
	2	0.00019528	0.02089512	e
Tyrosine (D014443)	3	0.00085140	0.03746136	d
Pregnancy in Diabetics (D011254)	1	0.00703107	0.03515533	d
	1	0.00730629	0.03653145	e
Hyperglycemia (D006943)	1	0.01748688	0.04066230	d
	1	0.01816762	0.04223941	e

^a^Benjamini & Hochberg procedure for controlling false discovery rate (FDR) [40]

^b^Analysis considering dataset of: (a) 232 genes annotated in cROH of pools shared between at least 12 animals of both generations; (b) 71 genes annotated in cROH of pools shared exclusively between 7th animals; (c) 1318 genes annotated in cROH of pools shared between at least four 16th animals more than 7th’s; (d) 56 genes annotated in F_ST_ windows of SNP data (FST ≥ 0.3); and (e) 60 genes annotated in FST windows of INDEL data (FST ≥ 0.3)

### Selection signatures in overlap with QTL in the TT line

**Fig. 7:**
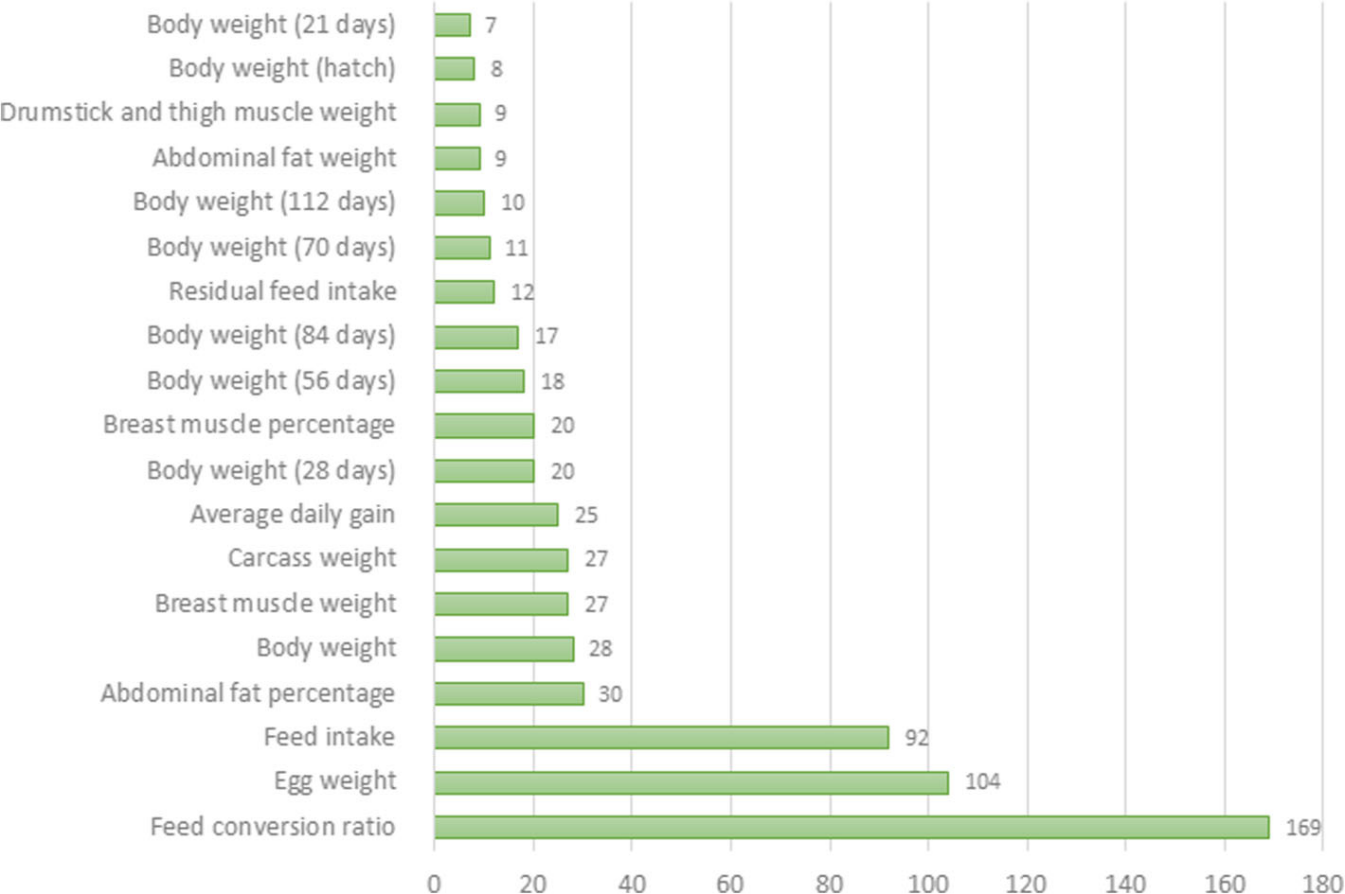
Manhattan plot of genome wide distribution of F_ST_ windows for INDEL dataset. Red line represents threshold of 0.3, windows above this value were considered candidate selection signatures

**Fig. 8:**
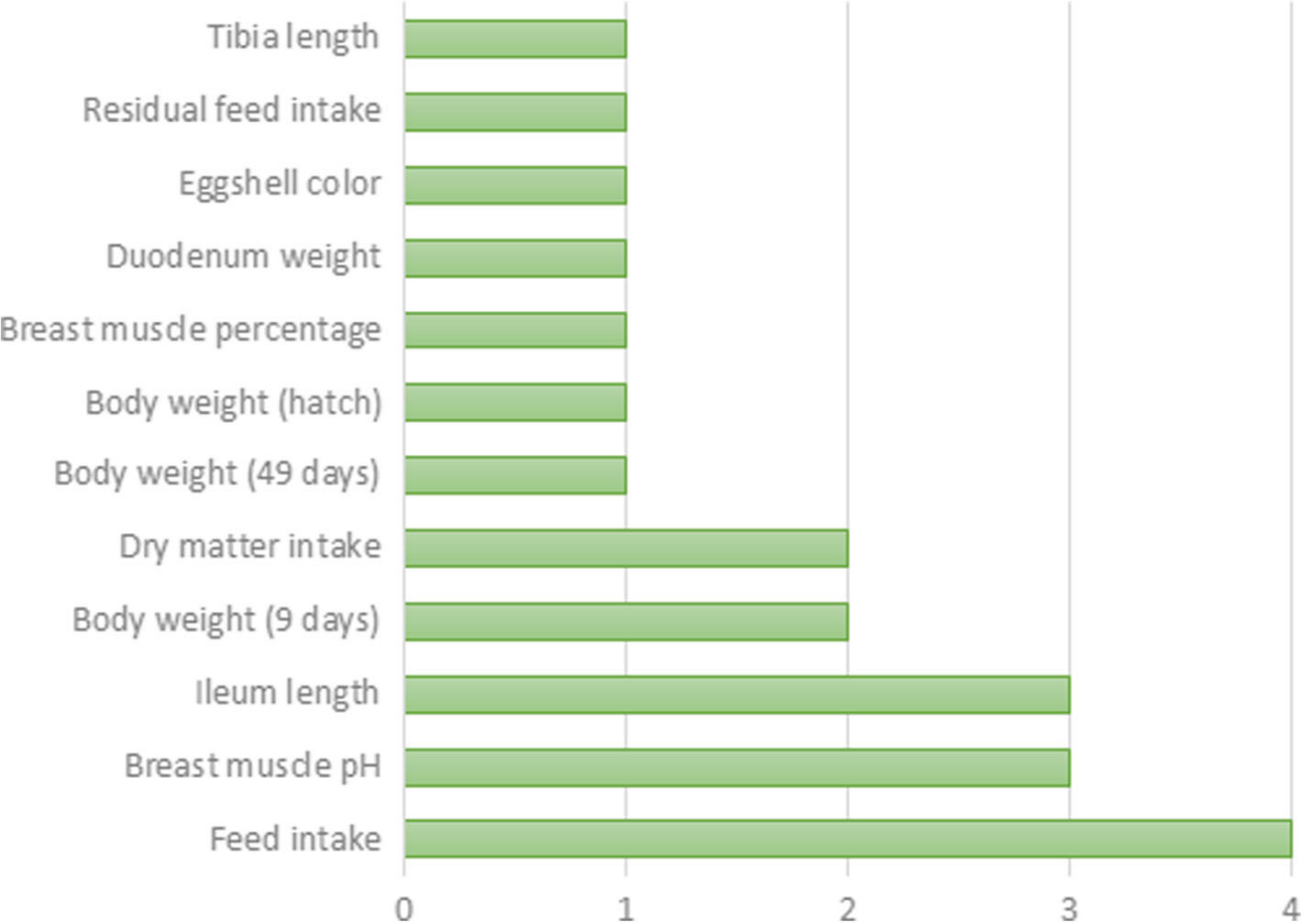
Manhattan plot of genome wide distribution of F_ST_ windows for INDEL dataset. Red line represents threshold of 0.3, windows above this value were considered candidate selection signatures

**Fig. 9:**
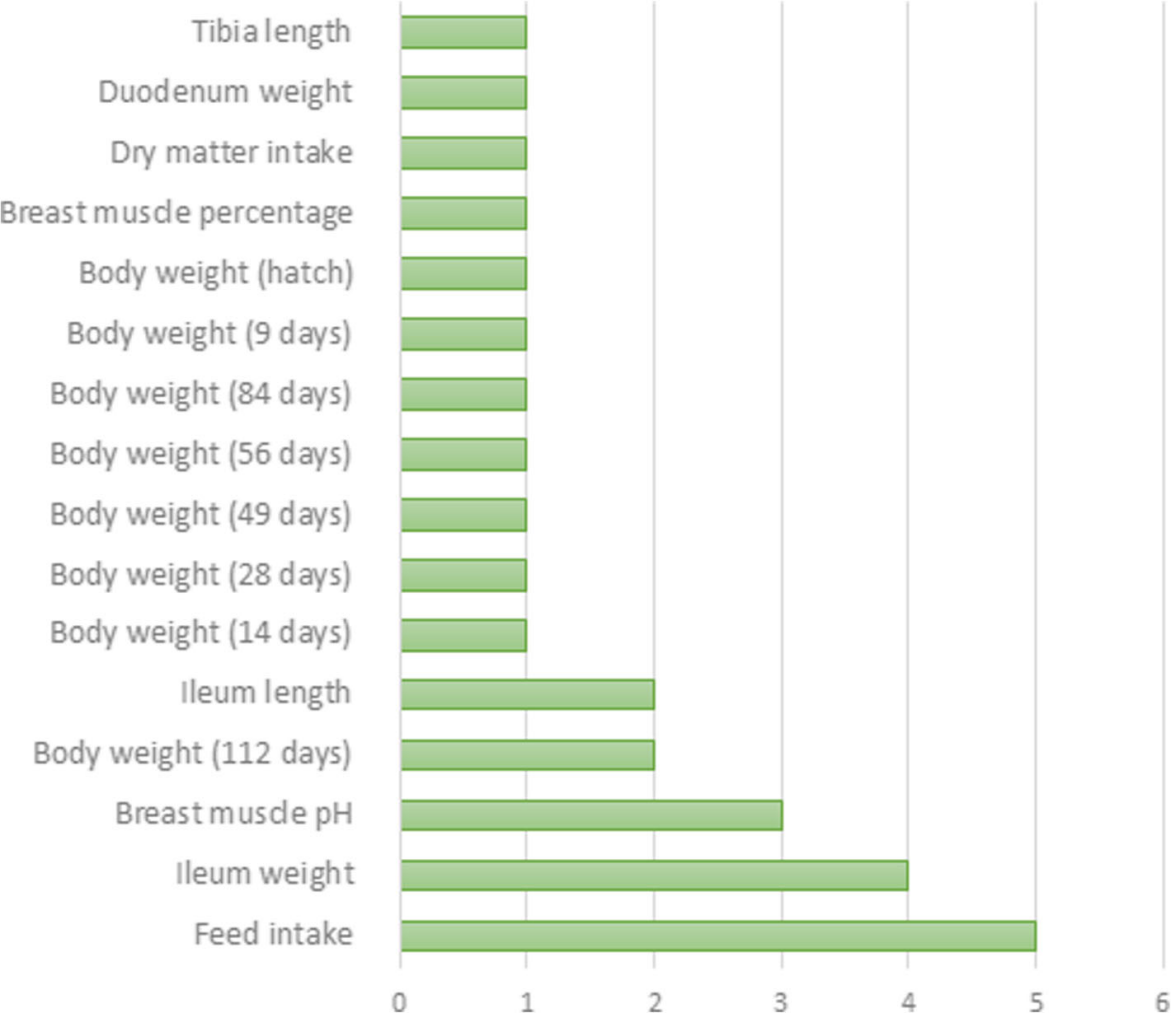
Manhattan plot of genome wide distribution of F_ST_ windows for INDEL dataset. Red line represents threshold of 0.3, windows above this value were considered candidate selection signature

To identify selection signatures associated with quantitative traits, we investigated the overlap between the regions of cROH, F_ST_ SNP and INDEL windows (≥ 0.3) with QTL regions previously associated with traits of economic interest in chickens available at the Chicken QTL database (release 37). About 72.8% of the 1941 cROH overlapped with QTL regions (*n* = 2617). There were also overlap of 60.1% of the 178 F_ST_ SNP windows and 68.2% of the 154 F_ST_ INDEL windows with QTL regions (*n* = 107 and 105, respectively). These QTL regions were associated with 143 different traits (Additional file 12) of which some are very important for broilers’ breeding program goals such as feed conversion rate, feed intake, average daily gain, body weight, breast muscle weight, and others (Figs. 7, 8 and 9).

We also identified cROH regions that overlapped with 10 QTL previously mapped for fatness traits on GGA5, GGA9, GGA10, GGA13, GGA15, and GGA27 in the same population utilized herein (TT Reference Population) (Table 6) [42]. There was no overlap between the F_ST_ windows (≥ 0.3) and these QTLs mapped for fatness.

**Table 6:** QTLs associated with fat traits in TT Reference Population overlapping with consensus runs of homozygosity (cROH)

Trait (QTL ID)^a^	Chr	QTL position (start-end)^b^	cROH position (start - end)
ABFW (160520)	5	38,000,437 – 38,996–916	38,015,470 – 38,234,917
			38,425,606 – 38,473,340
ABFW (160521)	10	7,000,336 – 7,998,549	6,978,426 – 7,049,244
			7,474,909 – 7,543,996
			7,931,784 – 7,932,642
ABFW (160522)	13	3,002,617 – 3,998,616	3,572,237 – 3,641,314
ABFP (160525)	5	38,000,437 – 38,996–916	38,015,470 – 38,234,917
			38,425,606 – 38,473,340
ABFP (160526)	10	7,000,336 – 7,998,549	6,978,426 – 7,049,244
			7,474,909 – 7,543,996
			7,931,784 – 7,932,642
ABFP (160527)	13	3,002,617 – 3,998,616	3,572,237 – 3,641,314
SKINW (160529)	15	6,000,311 – 6,999,944	6,175,697 – 6,362,475
			6,473,966 – 6,475,580
SKINW (160531)	24	5,000,105 – 5,999,010	4,720,727 – 5,060,139
			5,187,457 – 5,646,905
			5,899,715 – 5,962,715
SKINP (160534)	9	4,000,836 – 4,999,336	4,664,760 – 4,860,555
SKINP (160530)	15	6,000,311 – 6,999,944	6,175,697 – 6,362,475
			6,473,966 – 6,475,580

*ABFW* abdominal fat weight, *ABFP* abdominal fat percentage, *SKINW* skin weight, *SKINP* skin percentage

^a^QTLID from QTL chicken database

^b^Positions in the Gallus_gallus-5.0 version of the chicken genome

## Discussion

### Runs of homozygosity

ROH studies have addressed human evolution and diseases, conservation and evolution of wild species, and genomic features of livestock animals [2, 7, 13, 17]. The investigation of ROH as selection signatures using different generations of the TT line provided two new insights. First, the identification of important selection signatures that may refer to periods preceding the generations under study. Second, it allowed the comparison of how these selection signatures were shared among the individuals and how they have changed over the generations. Since shared ROH is an indication of regions under selection [18], the consensus regions of ROH (cROH) were used in this study to understand the possible biological consequences of selection in this broiler line.

The ROH identified in animals of TT line presented small to moderate sizes, ranging from 300 Kb to 4.9 Mb. Short ROH are most probable to be IBD genomic regions inherited from ancient ancestors indicating long term selection [43]. Over the generations, IBD segments tends to break down due to recombination events by repeated meiosis. Thus, ROH size is associated with the degree of shared parental ancestry and for how long it was passed across generations [32] and, as expected, short ROH regions shared between animals of the 7th and 16th generations encompassed genes associated with traits of interest (Additional file 10). It is important to mention that not all short ROH are IBD and a proportion of them may be identical-by-state (IBS) due to genetic drift, as well as population bottlenecks, and therefore some authors recommend caution in attributing these regions as candidate selection signatures [18, 44, 45]. The minimum size of 300 Kb was set in our analysis to detect ROH, considering that the use of WGS data calls ROH with smaller sizes compared to SNPchip data. A limitation in our study is that we were not able to determine which proportion of ROH is attributed to genetic drift and may lead to false positives. However, strategies were adopted to improve the chances to detect true selection signatures associated with the selection program. They were (i) using ROH regions in common with at least two animals, (ii) overlapping these regions with the Chicken QTL database, and (iii) performing MeSH overrepresentation analysis. These combined strategies reduce the chance to detect candidate selection signatures due to genetic drift.

In a study with offspring from animals of the 16th generation, Marchesi et al. [17] identified ROH in 1279 chickens using a high-density 600 K genotyping array data. They adopted the same parameters used in the present study, except for the minimum size of ROH and number of heterozygous SNP allowed. As expected, regions of ROH were commonly shared between both populations. More than 98% of the cROH identified herein in animals of the 16th generations, overlapped with ROH identified in the study of Marchesi et al. [17], corroborating our findings (Additional file 4). However, a higher number of ROH per animal and ROH with smaller sizes were identified in the 28 animals compared to those of Marchesi et al. [17]. We suggest that the reasons for identifying shorter ROH relies on the higher resolution of WGS data in comparison with SNPchip data. This difference in ROH calling was also observed in another study with feline that used both types of dataset for the same individuals [46].

It is also important to highlight that low coverage WGS data may present higher error rate of variant calling in comparison with SNPchip data, and this may lead to inaccuracy of ROH calling [29]. Thus, we followed parameters based on Ceballos et al. [29], which demonstrated equivalent results to SNPchip data’s results, when dealing with low coverage WGS. Moreover, in order to extend the chances of detecting accurate ROH, we opted to investigate consensus regions of ROH, i.e. regions of ROH in common with at least two animals, that might indicate regions under selection [18, 30]. All these observations corroborate with our suggestion that the smaller size and higher number of ROH possibly relies on the better resolution of WGS.

In our study, an increase in ROH abundance was observed, i.e. between the 7th and 16th generations there was an increase on the average number of ROH segments per animal and in the average size of segment per animal (Fig. 2 and Table 2). Investigation of the history of the breeding program indicate that these differences may have occurred due to a bottleneck effect. Marchesi et al. [17] estimated the N_e_ in TT line backing to 200 generations ago and reported a decay in N_e_, especially in the last five generations, ranging from 157 to 113 chickens (N_e_ of TT Reference Population). Thus, when a population size is reduced, the average of heterozygosity in a certain locus is expected to decline, depending on the N_e_ [47, 48]. The occurrence of a bottleneck effect is supported by the observed increase in the mean genomic inbreeding coefficient from the 7th to the 16th generation (7th F_ROH_ = 0.078 and 16th F_ROH_ = 0.202). It is worth to mention that, even if mating between close related individuals is avoided some level of inbreeding is unavoidable, because TT line is a closed population [17].

Furthermore, the percentage of animals sharing a ROH region increased from the 7th to the 16th generation (Fig. 4). Mastrangelo et al. [18] reported similar observations of an increase in the abundance of ROH in a sheep breed it and suggested that a decrease in the effective population size (N_e_) had occurred resulting in recent and historical autozygosity events. Thus, we suggest that the increase in homozygous regions across generations in TT line is consequence of selection pressure over genomic regions that are important to the breeding program’s goals jointly to reduction on the N_e_ and inbreeding. In fact, genes associated with traits of economic interest, such as the *APOB*, *POMC*, *PPARG* and other genes (Additional file 10), were annotated in regions shared with more animals of the 16th than with 7th generation, supporting that the regions containing these genes were under selection pressure in the respective period.

### F_ST_ windows

An alternative approach applied in this study for identification of selection signatures was the genetic differentiation method based on allele frequency differences called F_ST_ statistics. Previous studies have used this method for detecting selection signature in livestock species, such as broilers [14, 21, 22, 49, 50]. Here we compared two groups of animals of TT broiler line, 10 generations distant from each other. The parameters were the same used by Boschiero et al. [22], in the comparison of the TT line against a layer line. The authors states that windows of 20 Kb allow a finer resolution of the regions in addition to windows with sufficient number of markers, considering that the amount of variants in a window is essential for increasing the power of the analysis [22, 51]. This intent was achieved in our results since we obtained an average of 216 SNP/window and 18 INDEL/window, which were similar to the results obtained by Boschiero et al. [22] with averages of 268 SNP/window and 26 INDEL/window. Furthermore, there was a considerable number of windows in common between SNP and INDEL datasets with 87% of overlapping, a fact also observed by Boschiero et al. [22].

Estimates of F_ST_ range from zero, meaning no genetic difference between the subpopulations, up to 1.0, meaning complete genetic differentiation [52]. Although there is not a determined threshold to capture regions that indicate genetic differentiation as a candidate selection signature, some authors use a threshold for the top 0.1% values of F_ST_ [21, 22]. Here we established a threshold value of 0.3 in order to obtain regions that might be in a differentiation process in the TT line. Only 0.002% of the windows had F_ST_ values above this threshold, for SNP and INDEL datasets, and the highest estimated values were 0.598 and 0.555 for SNP and INDEL datasets, respectively. In addition, as discussed by Boschiero et al. [22], combining strategies to detect selection signatures minimizes the occurrence of false positives.

### Selection signatures of broiler performance and adaptation

Besides identifying regions in the chicken genome under selection pressure, knowing the genes annotated in these regions and how they biologically act is essential for understanding how the selection signatures contributed to the current phenotype of the evaluated animals. Since TT broiler line is under multi-trait selection since 1992 aiming to improve body weight, feed conversion, cut yields, breast weight, viability, fertility, and hatchability and to reduce abdominal fat [17, 23], it is expected that genes influencing the performance of these traits are under selection pressure.

Therefore, investigating which genes were annotated in the candidate selection signatures regions identified in both F_ST_ and ROH analysis helps to understand the biological mechanisms that affected the construction and evolution of the phenotype of TT line. In this sense, genes involved with traits of economic interest were identified in these regions. The genes *IGFB2, TGFB2, HOXD9, HOXD10, POMC SPP1, SPP2*, and *IGF1* were some of the genes annotated in the candidate selection signatures of TT line and that were previously found in other selection signatures and associated with traits such as growth, body weight and composition, abdominal fat, organogenesis and feed intake and consumption [22, 50, 53–66]. Furthermore, we identified a group of genes annotated in the selection signatures that are involved with structural constituents, cell differentiation, and development of muscle tissue: *ACTC1*, *AKAP6*, *ATP2A2*, *KCNMA1*, *MYO1B*, *MYO1C*, *MYO1E*, *MYO1F*, *MYO6*, *MYO7A*, *MYO10*, *MYO16*, *TPM4*, *VCL*, and *V1PR1* [22, 67]. Selection signatures identified in our analysis also indicate regions involved in lipid metabolism and adipose tissue development, encompassing the *ADCY2, AKAP6, APOB, ATPR2, IGFBP2, PLA2R1, PPARG, SCARB1* and *ZNF423* genes [22, 68–77].

Chickens raised in production systems are under several stressful conditions that can affect performance and the immune system of these animals [78, 79]. Stress challenged animals respond by changing their response behavior, metabolic rates, and functioning of cardiovascular and immune systems [78]. Thus, a selective pressure over genomic regions controlling responses to stressor conditions may occur, and the selection signatures identified with the ROH analysis shows a class of genes involved in these aspects: *ACE, BAG1, CACNA1C, ELP2, HSPA8, MOCOS, MRTO4, MYH9, NSUN2, PAX5, PQLC2* and *TRPM8* [16, 17, 80, 81].

### Changes in TT line across the generations

Enrichment analysis using MeSH was performed in order to provide a better integrated view of the changes that occurred. [82]. Adipose tissue was in overrepresentation among the genes of these regions, what is expected since selection for growth in broilers, could lead to elevated fat deposition [83]. In addition, overlaps of cROH regions with QTL associated with fat deposition in TT Reference population support that regions affecting these traits were indirectly selected across generations in TT line. Hyperglycemia was also overrepresented and it is possibly a consequence of fat deposition in these animals. The excess of adipose tissue in chickens may lead to a condition similar to the early stage of type 2 diabetes in humans, manifesting hyperglycemia and exogenous insulin resistance [84, 85]. Another overrepresentation was ‘pregnancy in diabetics’, and, as it is known, pregnancy is not a biological mechanism of birds. However, Nadaf et al. [83] discuss that some QTL associated with chicken fatness have genes playing a role in obesity and diabetes in humans, and since MeSH is a tool that comprises animals in general, this association may have be done due to genes with similar functions.

Cystatins was among genes annotated in regions that underwent allele frequency changes during the 7th and 16th generations. Cystatin is a superfamily of reversible competitive inhibitors of cysteine proteases such as calpains, cathepsins, and ficins, and the cystatin system have important roles in protein turnover, antigen presentation and disease immunity [86, 87]. As well, tyrosine was overrepresented among the genes of F_ST_ SNP windows, which is considered a nonessential amino acid in animals [88].

The ROH analysis also provided information about regions that may indicate selection in a period that precedes the studied generations. In these regions there were overrepresentation of genes involved in skeletal muscle and the matrilin proteins, both important for the growth of chickens. Matrilin is a four-member family of proteins composing extracellular matrix of some tissues as cartilage, a connective tissue. They bind to collagen-containing fiber and other matrix constituents and can form oligomers [89].

Furthermore, the abundant amount of overlaps between cROH and F_ST_ windows and QTL regions associated with traits of economic interest in broilers, such as feed conversion, feed intake, growth, and abdominal fat, enforces the results of candidate selection signatures involved in performance traits. This fact together corroborates that TT line have been selected for growth and muscle deposition for a long period with a possible consequence of increased fat deposition, and for some traits such as proteic turnover and metabolism of tyrosine.

## Conclusion

Regions under selection pressure in a paternal broiler line were investigated in this study. Using ROH analysis, we were able to identify regions that were inherited backing to common ancestors since the beginning of the broiler line origin, how these regions were shared between the animals of both generations, and what has changed in the genetic make-up of the TT line by selection between the 7th and 16th generations. F_ST_-based analysis revealed regions that changed between generations. Annotation and enrichment analysis revealed the selection program affected genes and biological processes involved in skeletal muscle, cartilage and adipose tissues development. The investigation of selection signatures provided valuable insights about genes and biological processes involved in performance, adaptation and disease traits.

## Additional files

Additional file 1: A text file with information of the 5721 ROH identified in the analysis with the 28 chickens. Information comprises individual identification (IID), chromosome (CHR), start (POS1) and end (POS2) positions, size (KB), number of SNP in each ROH (NSNP), and density of SNP (DENSITY). (TXT 256 kb)

Additional file 2: A compressed file of images (TIFF format) of genome wide distribution of runs of homozygosity (ROH) in TT population for each chromosome. (RAR 144 kb)

Additional file 3: A text file with information about pools of overlapping ROH among the 28 chickens. Information comprises pool identification (POOL), family identification (FID), individual identification (IID), chromosome (CHR), start (BP1) and end (BP2) positions, size (KB), and number of SNP in each ROH of in the pool. Each pool has *n + 2* lines: the *n* lines are respective to the *n* individuals in overlap and the last two represents the union and consensus regions of the overlap. (TXT 776 kb)

Additional file 4: A text file with information of F_ST_ windows using SNP dataset. Information comprises chromosome (CHR), start (START) and end (END) positions, number of variants (NVAR), and weighted F_ST_ values (FST) for each window. (XLSX 19935 kb)

Additional file 5: A text file with information of F_ST_ windows using INDEL dataset. Information comprises chromosome (CHR), start (START) and end (END) positions, number of variants (NVAR), and weighted F_ST_ values (FST) for each window. (TXT 2830 kb)

Additional file 6: List of the 5681 genes annotated in the 1941 consensus regions of runs of homozygosity (cROH). (TXT 2578 kb)

Additional file 7: List of the 56 genes annotated in the 178 FST windows (≥0.3) using SNP dataset. (XLS 5840 kb)

Additional file 8: List of the 60 genes annotated in the 154 FST windows (≥0.3) using INDEL dataset. (XLS 79 kb)

Additional file 9: **Table S1:** Genes annotated commonly between selection signatures of two or more datasets. The datasets were the 1941 consensus regions of ROH (cROH), the F_ST_ SNP windows (> 0.3), and the F_ST_ INDEL windows (> 0.3). (XLS 83 kb)

Additional file 10: **Table S2:** Genes previously associated with traits of interest located in candidate selection signatures. Those located in consensus regions of ROH presents the number of animals sharing the common region, and those located in F_ST_ windows present the respective weighted F_ST_ values. (XLSX 394 kb)

Additional file 11: positional information of overlaps between QTL from the QTL database with candidate selection signatures of ROH and F_ST_ windows (SNP and INDEL datasets). (DOCX 20 kb)

Additional file 12: positional information of overlaps between cROH of animals of the 7th and 16th generations with ROH of animals of the TT Reference Population. (XLSX 1198 kb)

## Data Availability

All data generated from the analyses of this work are public and included in this article in the main manuscript or as additional files. All the SNPs utilized were submitted to European Variation Archive (EVA) – EMBL-EBI, accession PRJEB25004 and to dbSNP (NCBI) with the submitter handle “LBA_ESALQ”. Additional datasets and scripts used in the analyses are available from the corresponding author under reasonable request.

## Abbreviations

ABFP: abdominal fat percentage
ABFW: abdominal fat weight
CEUA: Ethics Committee on Animal Utilization
CONCEA: National Council of Animal Experimentation Control
cROH: consensus region of runs of homozygosity
EHH: extended haplotype homozygosity
FDR: false discovery rate
F_ROH_: inbreeding coefficient based on runs of homozygosity
F_ST_: fixation index
IBD: identical-by-descent
IBS: identical-by-state
iHS: integrated haplotype score
INDEL: insertions and deletions
N_e_: effective population size
QTL: quantitative trait loci
ROH: runs of homozygosity
SKINP: skin percentage
SKINW: skin weight
SNP: single nucleotide polymorphism
UCSC: University of California, Santa Cruz
uROH: union region of runs of homozygosity
WGS: whole genome sequencing
