# Supplementary material for: Identification of selection signatures involved in performance traits in a paternal broiler line
Source: BMC Genomics. 2019 Jun 3;20:449. doi: 10.1186/s12864-019-5811-1 (PMC6547531; doi:10.1186/s12864-019-5811-1)
Supplement: Supplementary file 11 — positional information of overlaps between QTL from the QTL database with candidate selection signatures of ROH and FST windows (SNP and INDEL datasets). (DOCX 20 kb) [file 12864_2019_5811_MOESM11_ESM.docx]

**Identification of selection signatures involved in performance traits in a paternal broiler line**

Octávio Augusto Costa Almeida, Gabriel Costa Monteiro Moreira, Fernanda Marcondes de Rezende, Clarissa Boschiero, Jane de Oliveira Peixoto, Adriana Mercia Guaratini Ibelli, Mônica Corrêa Ledur, Luiz Lehmann Coutinho

**Supplementary Table 2** Genes previously associated with traits of interest located in candidate selection signatures

| Chr | Gene | Gene position | Candidate selection signature | Candidate selection signature position | n animals TT98/TT2007^a^ | FST weighted value |
| --- | --- | --- | --- | --- | --- | --- |
| 1 | MYH9 | 51,802,991 – 51,870,983 | F_ST_ INDEL window | 51,870,001 – 51,890,000 | - | 0.3158 |
| 1 | IGF-I | 55,335,204 – 55,383,631 | cROH | 55,149,208 – 55,359,089 | 7/13 | - |
| 1 | CACNA1C | 61,471,636 – 61,671,542 | cROH | 61,493,137 – 61,582,897 | 0/3 | - |
| 1 | ITPR2 | 67,751,004 – 67,983,940 | cROH | 67,475,528 – 67,977,445 | 2/7 | - |
| 1 | MYO1C | 138,120,025 – 138,390,526 | cROH | 137,959,482 – 138,138,743  138,355,619 – 138,459,726 | 2/4  4/5 | - |
| 1 | MYO7A | 193,221,562 – 193,279,711 | cROH | 193,062,724 – 193,346,675 | 2/1 | - |
| 2 | VIPR1 | 1,742,703 – 1,846,900 | cROH | 1,745,421 – 1,863,999 | 1/4 | - |
| 2 | MYO10 | 75,715,508 – 75,869,282 | cROH | 75,651,510 – 75,752,520 | 5/14 | - |
| 2 | ADCY2 | 79,496,841 – 79,702,592 | cROH | 79,332,597 – 79,659,423 | 2/5 | - |
| 2 | NSUN2 | 80,034,291 – 80,050,765 | cROH | 79,940,076 – 80,187,400 | 2/4 | - |
| 2 | MOCOS | 85,073,411 – 85,199,421 | cROH | 84,971,233 – 85,451,910 | 3/1 | - |
| 2 | BAG1 | 85,906,463 – 85,915,694 | cROH | 85,485,648 – 85,943,053 | 2/2 | - |
| 2 | ELP2 | 88,712,300 – 88,744,632 | cROH | 88,409,172 – 88,718,424  88,718,448 – 88,734,342 | 2/1  2/1 | - |
| 3 | MYO6 | 80,736,607 – 80,807,004 | cROH | 80,805,954 – 80,905,165 | 2/3 | - |
| 3 | MATN3 | 101,853,568 – 101,868,845 | cROH | 101,659,552 – 101,995,693 | 1/2 | - |
| 3 | APOB | 102,659,050 – 102,693,303 | cROH | 102,641,927 – 102,729,608 | 1/5 | - |
| 3 | POMC | 105,818,021 – 105,834,013 | cROH | 105,658,152 – 106,099,077 | 1/2 | - |
| 5 | CCND1 | 17,525,970 – 17,539,612 | F_ST_ SNP window  F_ST_ INDEL window | 17,530,001 – 17,550,000  17,530,001 – 17,550,000 | - | 0.3103  0.3544 |
| 5 | ACTC1 | 32,283,463 – 32,288,278 | cROH | 32,232,519 – 32,293,369 | 5/8 | - |
| 5 | AKAP6 | 34,843,920 – 35,050,252 | cROH | 34,751,946 – 34,917,590 | 2/7 | - |
| 6 | KCNMA1 | 13,362,771 – 13,800,472 | cROH | 13,160,512 – 13,373,205  13,532,853 – 13,536,574  13,537,312 – 13,573,098  13,573,470 – 14,246,233 | 2/3  2/3  2/3  2/3 | - |
| 6 | VCL | 8,014,556 – 8,105,794 | cROH | 8,136,748 – 8,477,976  8,783,567 – 9,165,313 | 1/3  0/3 | - |
| 7 | TRPM8 | 5,574,232 – 5,604,112 | F_ST_ SNP windows  F_ST_ INDEL windows | 5,560,001 – 5,580,000  5,570,001 – 5,590,000  5,580,001 – 5,600,000  5,590,001 – 5,610,000  5,600,001 – 5,620,000  5,570,001 – 5,590,000  5,580,001 – 5,600,000  5,590,001 – 5,610,000  5,600,001 – 5,620,000 | - | 0.3003  0.3227  0.3396  0.3371  0.3113  0.3044  0.3080  0.3206  0.3490 |
| 7 | SPP2 | 5,611,450 – 5,619,226 | F_ST_ SNP windows  F_ST_ INDEL windows | 5,600,001 – 5,620,000  5,610,001 – 5,630,000  5,600,001 – 5,620,000  5,610,001 – 5,630,000 | - | 0.3490  0.4495  0.3113  0.35.86 |
| 7 | MYO1B | 8,014,556 – 8,105,794 | cROH | 7,849,666 – 8,160,956 | 8/5 | - |
| 7 | HOXD9 | 16,362,036 – 16,363,342 | cROH | 15,889,809 – 16,664,514 | 0/2 | - |
| 7 | HOXD10 | 16,367,140 – 16,369,673 | cROH | 15,889,809 – 16,664,514 | 0/2 | - |
| 7 | PLA2R1 | 21,941,750 – 21,977,517 | cROH | 21,726,831 – 21,964,077 | 0/5 | - |
| 7 | IGFBP2 | 23,382,780 – 23,435,025 | cROH | 23,428,863 – 23,483,121 | 4/3 | - |
| 10 | MYO1E | 6,672,861 – 6,750,075 | cROH | 6,643,521 – 6,674,804 | 1/4 | - |
| 11 | ZNF423 | 6,819,994 – 7,018,204 | F_ST_ INDEL window | 6,960,001 – 6,980,000 | - | 0.3193 |
| 12 | PPARG | 4,859,854 – 4,880,521 | cROH | 4,774,152 – 4,978,240 | 1/4 | - |
| 15 | SCARB1 | 4,544,980 – 4,558,143 | cROH | 4,423,619 – 4,565,327 | 5/5 | - |
| 15 | ATP2A2 | 5,344,147 – 5,385,167 | cROH | 5,346,615 – 5,480,296 | 3/7 | - |
| 19 | MYO1C | 5,189,500 – 5,240,189 | cROH | 5,054,141 – 5,419,934 | 1/5 | - |
| 21 | MRTO4 | 4,666,392 – 4,668,550 | cROH | 4,532,882 – 4,841,708 | 1/1 | - |
| 21 | PQLC2 | 4,672,913 – 4,677,272 | cROH | 4,532,882 – 4,841,708 | 1/1 | - |
| 24 | HSPA8 | 3,073,404 – 3,080,079 | cROH | 2,904,030 – 3,095,369 | 1/3 | - |
| 27 | ACE | 2,830,975 – 2,847,223 | cROH | 2,523,474 – 2,834,249 | 1/1 | - |
| 28 | MYO1F | 1,741,275 – 1,754,399 | cROH | 1,723,513 – 1,789,434 | 0/4 | - |
| 28 | TPM4 | 4,446,782 – 4,454,984 | cROH | 4,428,153 – 4,621,310 | 7/5 | - |

Chr = chromosome

**^a^**Number of animals of TT98 and TT2007 with ROH overlapping the same region.

Annotation in BioMart Ensembl (Ensembl Genes 94) using the current chicken genome version (Gallus_gallus-5.0, UCSC)
